# A multicenter retrospective analysis of clinical outcomes of intracranial chondrosarcoma in 26 patients

**DOI:** 10.1038/s41598-023-41378-w

**Published:** 2023-09-05

**Authors:** Hongyuan Liu, Zongping Li, Yafei Xue, Tianzhi Zhao, Yingxi Wu

**Affiliations:** 1 Department of Neurosurgery, Mianyang Central Hospital, School of Medicine, University of Electronic Science and Technology of China, Mianyang, Sichuan People’s Republic of China; 2 Department of Neurosurgery, Tangdu Hospital, Air Force Medical University, Xi’an, Shaanxi People’s Republic of China

**Keywords:** Neurology, Oncology

## Abstract

Intracranial chondrosarcoma is a rare tumor with limited reports. We reviewed the clinical outcomes, imaging findings, and pathological characteristics at three centers to improve the diagnosis and treatment of intracranial chondrosarcoma. We retrospectively analyzed 26 patients with intracranial chondrosarcoma who had undergone surgical treatment at Tangdu Hospital of Air Force Military Medical University, Mianyang Central Hospital, and Nanchong Central Hospital from January 2010 to July 2022. Clinical manifestations, imaging features, surgical treatment, prognosis, and overall survival (OS) were analyzed. All 26 chondrosarcomas were located at the skull base. Gross total resection (GTR), subtotal resection (STR), and partial resection (PR) were performed in 14, 10, and 2 cases, respectively. Four cases underwent endoscopic transnasal surgery, while the remaining cases underwent craniotomy. The clinical symptoms were evaluated 1 week after surgery, and 15 cases were relieved to varying degrees. Postoperative complications included pulmonary infection, subcutaneous hydrops, dysphagia and choking, facial numbness, abducens paralysis, and intracranial infection (ICI). Fifteen cases received postoperative adjuvant radiotherapy. Seven cases showed recurrence: two with PR, four with STR, and one with GTR. Six cases received reoperation or radiotherapy after tumor progression, and one untreated patient died 5 months after tumor recurrence. The extent of tumor resection (HR 21.74, 95% CI 1.25–376.6, *P* = 0.03) and pathological grading (HR 131.99, 95% CI 4.05–4300.5, *P* = 0.006) were associated with improved OS. We presented our experience in the treatment of intracranial chondrosarcoma at three centers in the past 12 years. Intracranial chondrosarcoma lacked typical imaging features and are difficult to differentiate from other skull base lesions. Maximum extent of tumor resection with minimal injury to neurological function remains the most important treatment strategy. The extent of surgical resection and pathological grading were found to be predictors for OS.

## Introduction

Intracranial chondrosarcoma is an extremely rare tumor of the central nervous system (CNS) that accounts for 0.15% of all intracranial tumors^1^. Most intracranial chondrosarcomas occur at the skull base and originate from cartilage tissue; however, cases originating from non-cartilage tissue, such as the fourth ventricle and brain parenchyma, have also been reported^2–4^. The clinical manifestations of chondrosarcoma vary depending on the intracranial location, and may include tinnitus, facial numbness, diplopia, and decreased vision. Head computed tomography (CT) and magnetic resonance imaging (MRI) help diagnose chondrosarcoma, but it is difficult to differentiate chondrosarcoma from other tumors^5,6^. Intracranial chondrosarcomas have primarily been reported as case reports, are often difficult to characterize, and have a high misdiagnosis rate. To improve the diagnosis and treatment of intracranial chondrosarcoma, we reviewed 26 cases of intracranial chondrosarcoma treated with surgery; summarized the clinical features, imaging findings, surgical methods, and pathological characteristics; and put forward an optimum treatment planning for chondrosarcoma to achieve satisfactory results.

## Methods

This study was approved by the local ethics committee of the Tangdu Hospital of Air Force Military Medical University (AFMU), Mianyang Central Hospital (MCH) and Nanchong Central Hospital (NCH). All methods were performed in accordance with relevant guidelines and regulations. The study was retrospective, written informed consent could not be received from all patients or guardians. Requirement of the informed consent was waved by the three ethics committee.

A total of 26 patients were diagnosed with intracranial chondrosarcoma by postoperative pathology and received surgical treatment at Tangdu Hospital of AFMU (Xi’an, China), MCH (Mianyang, China) and NCH (Nanchong, China) between March 2010 and July 2022. We collected information on age, gender, clinical symptoms, imaging manifestations, extent of tumor resection, pathological results, postoperative complications, adjuvant radiotherapy, recurrence, and survival. Preoperative and postoperative CT or MRI were performed to determine the extent of tumor resection. Preoperative CT angiography (CTA), magnetic resonance angiography (MRA), and magnetic resonance venography (MRV) were used to evaluate chondrosarcoma in relation to blood vessels and venous sinuses. The degree of surgical resection included the following three options: gross total resection (GTR), no tumor residue; subtotal resection (STR), > 90% of tumor removed; and partial resection (PR), 50–90% of tumor removed^7^. Follow-up was conducted every 3–6 months by outpatient or telephone interview. Clinical symptoms and imaging findings were assessed after 6 months.

### Statistical analysis

Descriptive statistics were presented to summarize data related to demographics, surgical access, extent of tumor resection, tumor grade, adjuvant radiotherapy, and tumor volume. Univariate analysis and multivariate logistic regression were used to predict the factors of recurrence. To determine the factors associated with overall survival (OS), Cox proportional risk model was constructed. The model was first validated to select covariates, and then reverse selection was performed using *P* ≥ 0.05 as the removal criterion. Statistical analysis was performed using 4.2.1 (https://www.r-project.org). Survival curve was plotted by Kaplan–Meier. *P* value < 0.05 was considered statistically significant.

## Results

### Clinical features

The clinical features of the reported cases are summarized in Table 1. There were 16 females and 10 males, with a mean age of 46.2 ± 11 years (range, 28–68 years). The duration of symptoms ranged from 0.01 to 120 months (mean, 19.05 ± 33.2 months). Preoperative symptoms included headache (9/26), vision loss (9/26), hearing loss (4/26), blepharoptosis (3/26), facial numbness (2/26), dizziness (2/26), diplopia (2/26), facial numbness (2/26), abducens paralysis (2/26), hemiparesis (1/26), and hoarseness (1/26). Headache and vision loss were the most common symptoms in the study. The possible cause of headache was increased intracranial pressure (ICP) due to large space-occupying lesions or tumor stimulation of the dura. Compression of the optic nerve, abducens nerve, posterior cranial nerves, trigeminal nerve, and/or oculomotor nerve resulted in decreased vision, diplopia, hoarseness, facial numbness, limited eye movement, and blepharoptosis.

**Table 1:** Patients treated for intracranial chondrosarcoma between January 2010 and July 2022.

Case #	Sex	Age (y)	Symptoms	Symptom duration (m)	Tumor site	Tumor volume (cm^3^)	Preoperative diagnosis	Surgical procedure	Surgical resection	Residual tumor volume (cm^3^)	Postoperative complications	Postoperative radiotherapy	Grade	Recurrence	Follow-up (m)
1	F	36	Headache	2	Cerebellum	15.36	Chondrosarcoma metastasis	Posterior sigmoid sinus	GTR	–	–	Yes	2	Yes	16
2	M	59	Headache	12	Mastoid apex, left side of atlas	45.47	Chondrosarcoma	Posterior midline	GTR	–	–	Yes	1	No	45
3	M	48	Facial numbness; diplopia	0.01	Parasellar	120	Meningioma	Expanded pterion	STR	3.2	Abducens paralysis; pulmonary infection	Yes	2	Yes	18
4	F	65	Headache; dizziness	6	Cerebellopontine angle	9.10	Schwannoma	Posterior sigmoid sinus	GTR	–	Facial numbness	No	1	No	72
5	M	52	Vision loss; hemiparesis	120; 0.67	Sella, parasellar middle cranial fossa	140	Chordoma	Orbitozygomatic	PR	41.74	Facial numbness	Yes	1	Yes	18
6	M	36	Abducens paralysis; blepharoptosis	36	Middle and posterior cranial fossa	262.8	Schwannoma	Expanded pterion	PR	103.12	Subcutaneous hydrops	Yes	1	Yes	19
7	F	59	Hoarseness	12	Middle cranial fossa, slope	165.82	Chordoma	Supratentorial and infratentorial	STR	8.57	–	Yes	1	No	51
8	F	61	Vision loss; blepharoptosis	72	Parasellar	43.99	Hemangioma	Orbitozygomatic	STR	2.4	–	Yes	1	Yes	42
9	F	31	Hearing loss	1	Cerebellopontine angle	23.81	Schwannoma	Posterior sigmoid sinus	GTR	–	Dysphagia and choking; pulmonary infection	No	1	No	27
10	F	36	Diplopia	0.25	Middle and posterior cranial fossa	74.78	Chondroma	Supratentorial and infratentorial	STR	5.19	Subcutaneous hydrops	Yes	1	No	15
11	M	42	Vision loss	1	Parasellar; middle cranial fossa	311.52	Chondrosarcoma	Expanded pterion	GTR	–	Pulmonary infection; eyelid ptosis; abducens paralysis; ICI	Yes	1	No	33
12	F	58	Hearing loss; facial numbness	12;0.5	Slope	45.36	Chordoma	EETA	STR	1.68	–	Yes	1	No	33
13	F	42	Headache	12	Craniocervical junction	48.54	Chordoma	Posterior median	GTR	–	–	No	1	No	15
14	F	33	Headache	12	Cerebellopontine angle	27.75	Schwannoma	Posterior sigmoid sinus	STR	0.54	Pulmonary infection	Yes	1	No	10
15	F	56	Headache; vision loss	120	Sella	14.4	teratoma	EETA	GTR	–	–	No	1	No	10
16	M	52	Hearing loss	24	Cerebellopontine angle	41.58	Schwannoma	Posterior sigmoid sinus	STR	2.28	Dysphagia and choking; pulmonary infection	No	1	Yes	40
17	M	39	Vision loss	6	Parasellar	18.41	Chondrosarcoma	Pterion	GTR	–	–	No	1	No	90
18	F	39	Vision loss	1	Parasellar	32.55	Pituitary adenoma	Pterion	STR	0.78	–	Yes	1	No	72
19	F	38	Hearing loss	3	Cerebellopontine angle	28.8	Schwannoma	Posterior sigmoid sinus	GTR	–	Dysphagia and choking; ICI	Yes	1	No	60
20	F	47	Headache; dizziness	12	Parasellar	18.63	Meningioma	Pterion	GTR	–	–	No	1	No	47
21	M	42	Vision loss	3	Parasellar	24.96	Meningioma	Pterion	GTR	–	Subcutaneous hydrops	No	1	No	27
22	F	38	Vision loss	4	Parasellar	56	Chordoma	EETA	STR	3.23	–	Yes	1	Yes	46
23	M	47	Headache	12	Posterior cranial fossa	20.74	Chordoma	Posterior sigmoid sinus	GTR	–	–	No	1	No	70
24	F	49	Abducens paralysis; blepharoptosis	1	Parasellar	48.36	Chondrosarcoma	Expanded pterion	GTR	–	Subcutaneous hydrops	No	1	No	60
25	M	28	Vision loss	1	Sella	8.97	Pituitary adenoma	EETA	GTR	–	–	Yes	1	No	52
26	F	68	Headache	7	Parasellar	45.76	Chondroma	Expanded pterion	STR	2.35	Pulmonary infection	Yes	1	No	29

*y* Year, *m* month, *ICI* intracranial infection, *EETA* endoscopic endonasal transsphenoidal approach, *GTR* gross total resection, *STR* subtotal resection, *PR* partial resection.

### Preoperative imaging and diagnosis

Preoperative images showing the location of the tumor are listed in Table 1. The scope of tumor invasion included parasellar region (11/26), posterior fossa (7/26), middle and posterior cranial fossa (3/26), middle and posterior cranial fossa (2/26), craniocervical junction (2/26), slope (2/26), and sella (2/26). One patient had a history of surgery for “spinal canal chondrosarcoma” and one had a recurrence of intracranial chondrosarcoma. The mean tumor volume was 65.13 cm^3^ (range, 8.97–311.52 cm^3^). CT imaging showed that almost all the bone around the lesions had erosions and destruction; a high–low mixed-density shadow was found in 15 cases and an irregular soft tissue shadow was found in 10 cases. Fifteen patients underwent CTA or MRA to determine the relationship between the tumor and surrounding blood vessels. On MRI, there were small speckled hypointensities or mixed signals with isometric or long T1 and T2 signals. According to preoperative imaging, one case was diagnosed with teratoma, one with hemangioma, two with chondroma, three with meningioma, six with schwannoma, and six with chordoma.

### Surgical outcomes

Surgical approach was selected according to the size and location of the tumor (Table 1). Tumors were removed by craniotomy in 20 cases and by endoscopic endonasal transsphenoidal approach in four cases. Craniotomy approaches mainly included pterion or expanded pterion approach (9/26), posterior sigmoid sinus approach (5/26), orbitozygomatic approach (2/26), paramedian approach (2/26), posterior median extension approach (2/26), and combined supratentorial and infratentorial approach (2/26). Intraoperatively, the tumor was found to be involved with the internal carotid artery (ICA) (8/26) and vertebral artery (2/26), and with the cranial nerves (CNs) II (4/26), III (4/26), V (3/26), VI (2/26), VII (2/26), VIII (2/26), and IX (1/26). GTR, STR, and PR were performed in 14, 10, and 2 cases, respectively. One week after surgery, headache was relieved in six cases, vision acuity improved in five cases, hearing improved in two cases, and facial numbness improved in one case.

### Postoperative complications

Postoperative complications included pulmonary infection (n = 6), subcutaneous hydrops (n = 4), dysphagia and choking (n = 3), facial numbness (n = 2), abducens paralysis (n = 2), intracranial infection (ICI) (n = 2), and eyelid ptosis (n = 1). There were no deaths due to postoperative complications.

Continuous lumbar cistern drainage and antibacterial treatment were used for ICI. Subcutaneous hydrops was cured by puncture and pressure bandaging. There was no case of dural repair to treat subcutaneous hydrops. Pulmonary infection was treated by sputum excretion and antibiotics. Bronchoscopic sputum aspiration and tracheotomy were feasible when pulmonary infection was serious. Nerve damage was treated by rehabilitation.

### Histopathology and immunohistochemistry

Pathological examination showed cartilage tissue proliferation forming lobulated structures, containing a large number of heteromorphic nuclei and chondrocytes, with double nucleus formation in some cells, and accompanied by irregular ossification, calcification, or mucus-like substances. Immunohistochemistry (IHC) was performed in 15 cases. S100 was positive in all cases; SOX-9 was positive in nine cases; and epithelial-membrane antigen (EMA) was negative. A total of 24 cases were diagnosed as chondrosarcoma (WHO I), and two cases were diagnosed as chondrosarcoma (WHO II).

### Recurrence and progression

Two cases had a history of “resection of intravertebral chondrosarcoma.” One case was treated with gamma-knife surgery (GKS) (peripheral dose, 14 Gy; central dose, 28 Gy) 1 month after surgery, and 15 cases received linear accelerator-assisted radiotherapy (total dose, 60 Gy). Seven cases showed recurrence, including two cases of PR, four cases of STR, and one case of GTR, i.e., two cases of grade 2 chondrosarcoma and five cases of grade 1 chondrosarcoma. After tumor recurrence and progression, one case received surgery and radiotherapy, two cases were treated with surgery alone, and three cases received radiotherapy alone. Five cases died between 6 and 22 months after treatment, and one untreated case died after 5 months.

### Statistical analysis of prognostic factors for OS

**Figure 1:**
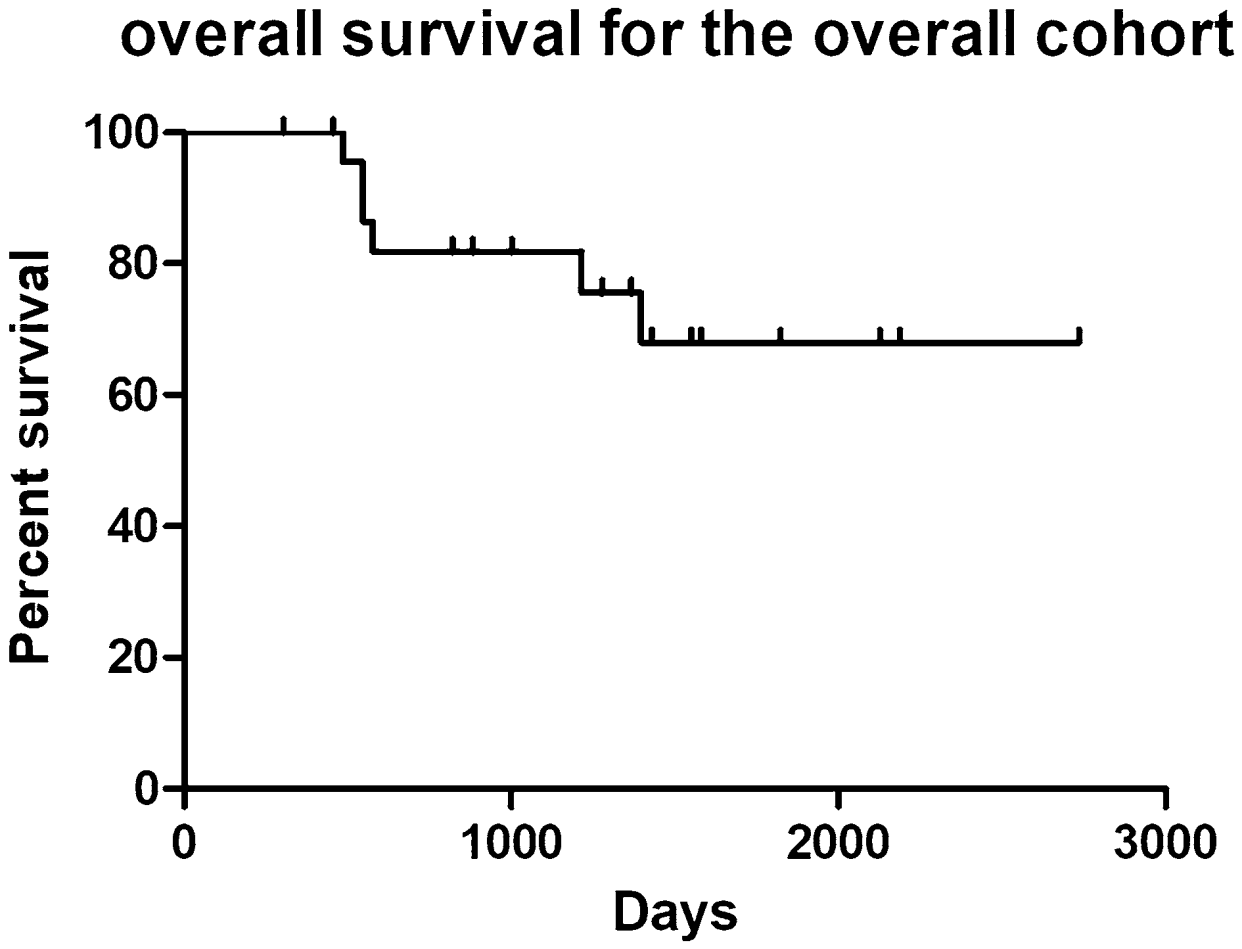
Kaplan–Meier OS curves for the overall cohort.

**Figure 2:**
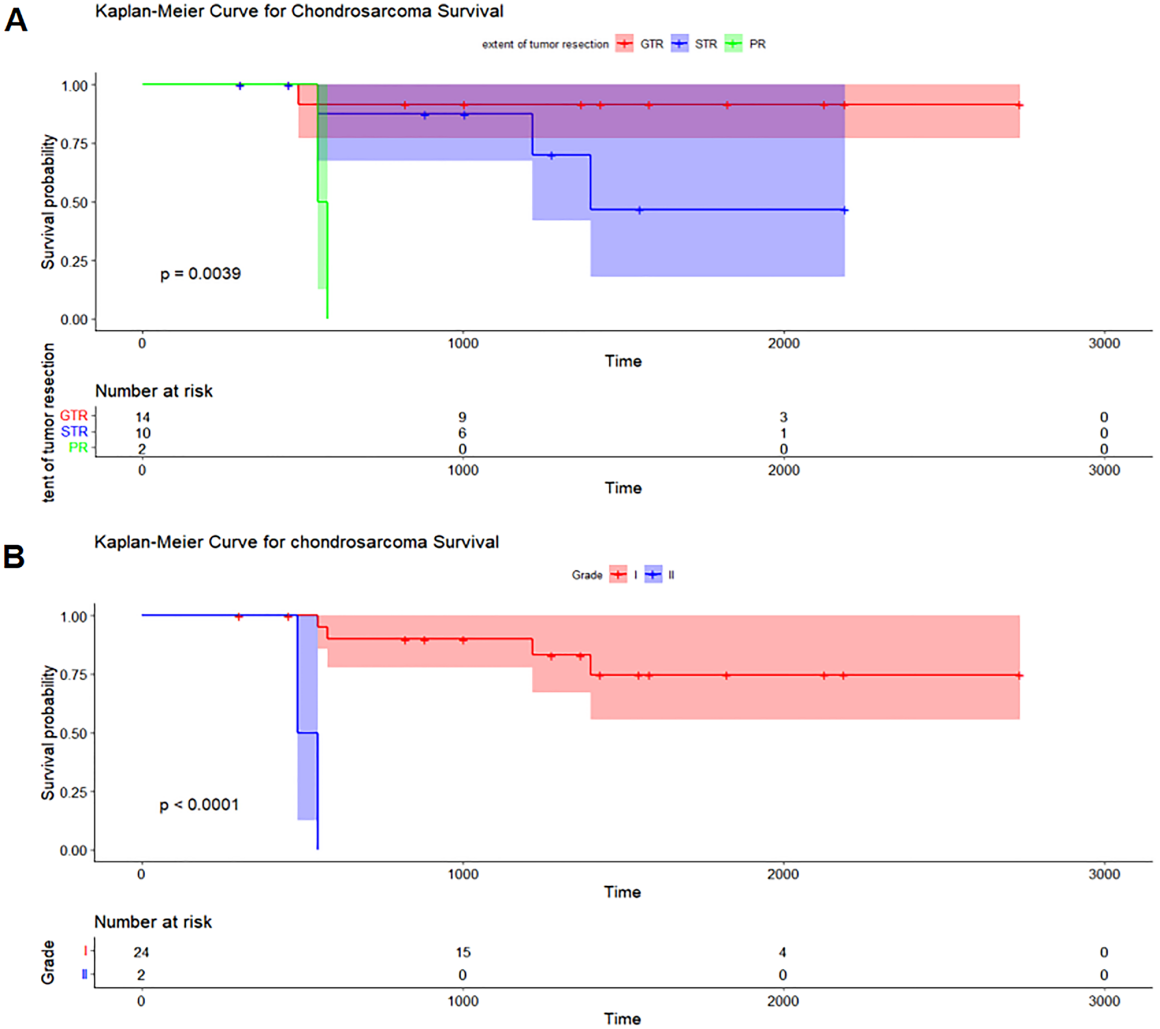
Kaplan–Meier curve shows the effect of tumor resection degree on the overall survival (OS) of the entire cohort (**A**). Kaplan–Meier curve shows the effect of tumor WHO grade on the OS of the entire cohort (**B**).

The mean follow-up time for the combined cohort (26 cases) ranged from 10 to 90 months (average, 39.12 months). Figure 1 displays the Kaplan–Meier OS curves for the overall cohort. The 1, 3 and 5-year OS were 100%, 81.8% (95% CI 67.2–99.6%) and 68% (95% CI 49.1–94.1%). Univariate Cox regression analysis—including sex, age, surgical approach, extent of tumor resection, adjuvant radiotherapy, tumor volume, and pathological grading—showed that extent of tumor excision (HR 5.7, 95% CI 1.5–22, *P* = 0.01) (Fig. 2A), tumor volume (HR 7, 95% CI 1.3–39, *P* = 0.03), and pathological grading (HR 42, 95% CI 3.5–500, *P* = 0.003) (Fig. 2B) affected the OS. Other factors—including sex (HR 2.9, 95% CI 0.52–16, *P* = 0.23), age (HR 0.97, 95% CI 0.9–1, *P* = 0.46), surgical approach (HR 0.93, 95% CI 0.11–8, *P* = 0.95), and adjuvant radiotherapy (HR 0.21, 95% CI 0.024–1.8, *P* = 0.15)—were not significant. Tumor grade, extent of tumor resection, and tumor volume were added to the multivariate Cox regression analysis. The extent of tumor resection (HR 21.74, 95% CI 1.25–376.6, *P* = 0.03) and pathological grading (HR 131.99, 95% CI 4.05–4300.5, *P* = 0.006) were protective factors for OS.

## Case illustration

### Case 2

**Figure 3:**
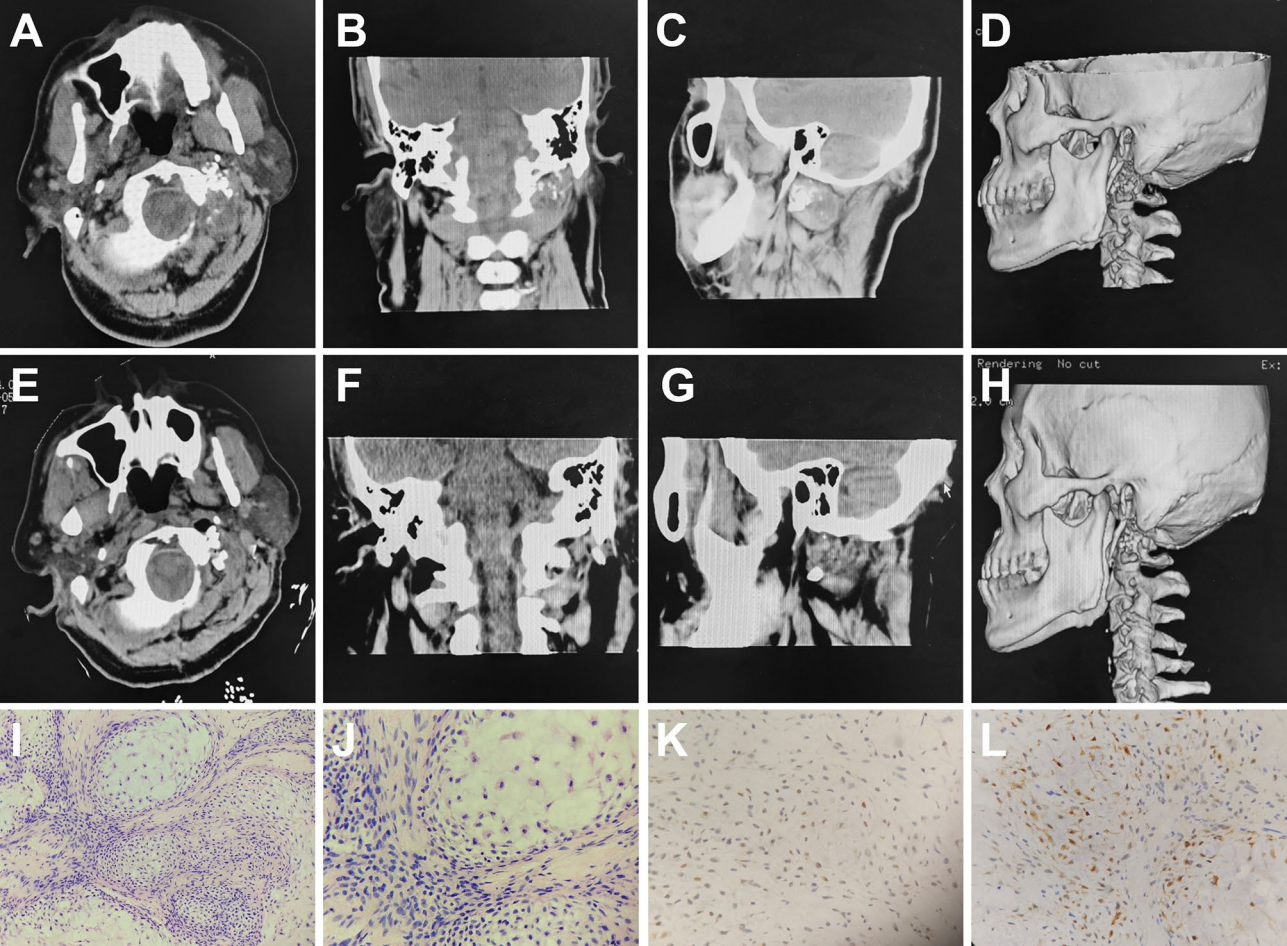
Comparison of preoperative and postoperative CT and histopathological examination. Preoperative plain CT scanning and three-dimensional reconstruction show osteolytic destruction in the left lateral mass of the atlas and a surrounding soft tissue mass (**A**–**D**). Postoperative plain CT scanning and three-dimensional reconstruction demonstrate that the tumor was removed (**E**–**H**). Histopathological examination at 200 × and 400 × magnification shows that chondrosarcoma cells are densely arranged with lobulated structure (**I**,**J**). Immunohistochemical staining of SOX-9 (+, **K**) and S-100 (+, **L**), at 400 × magnification.

A 59-year-old man was admitted with retroauricular pain on the left side lasting for 1 year. He had a history of hypertension for more than 10 years and oral medication use for blood pressure control, and a history of diabetes for more than 10 years with insulin use for blood glucose control. Coronary artery stenting was performed in 2013 and 2016. The patient had a history of allergy to levofloxacin and iodine. There were no positive signs on neurological examination. MRI was not performed on this patient because of the history of coronary artery stenting. CT suggested bone destruction of the mastoid apex and left side of the atlas with soft tissue mass formation (Fig. 3A–D). The preoperative diagnosis was chondrosarcoma. The patient was placed in a prone position, and an incision was made at the posterior midline and leftward turning with the lowest axial spinous process and the highest occipital tuberosity. A white tumor with a hard texture and scarce blood supply was seen behind the mastoid process. The tumor was removed, and the vertebral artery was well-preserved during the surgery. The patient's symptoms disappeared after surgery, and CT indicated total tumor resection (Fig. 3E–H). Pathological examination showed that the cells were densely arranged in lobular structure (Fig. 3I,J). Immunohistochemistry showed positivity for SOX-9 and S-100 (Fig. 3K,L). Postoperative radiotherapy was administered. There was no recurrence during 45 months of follow-up.

### Case 11

**Figure 4:**
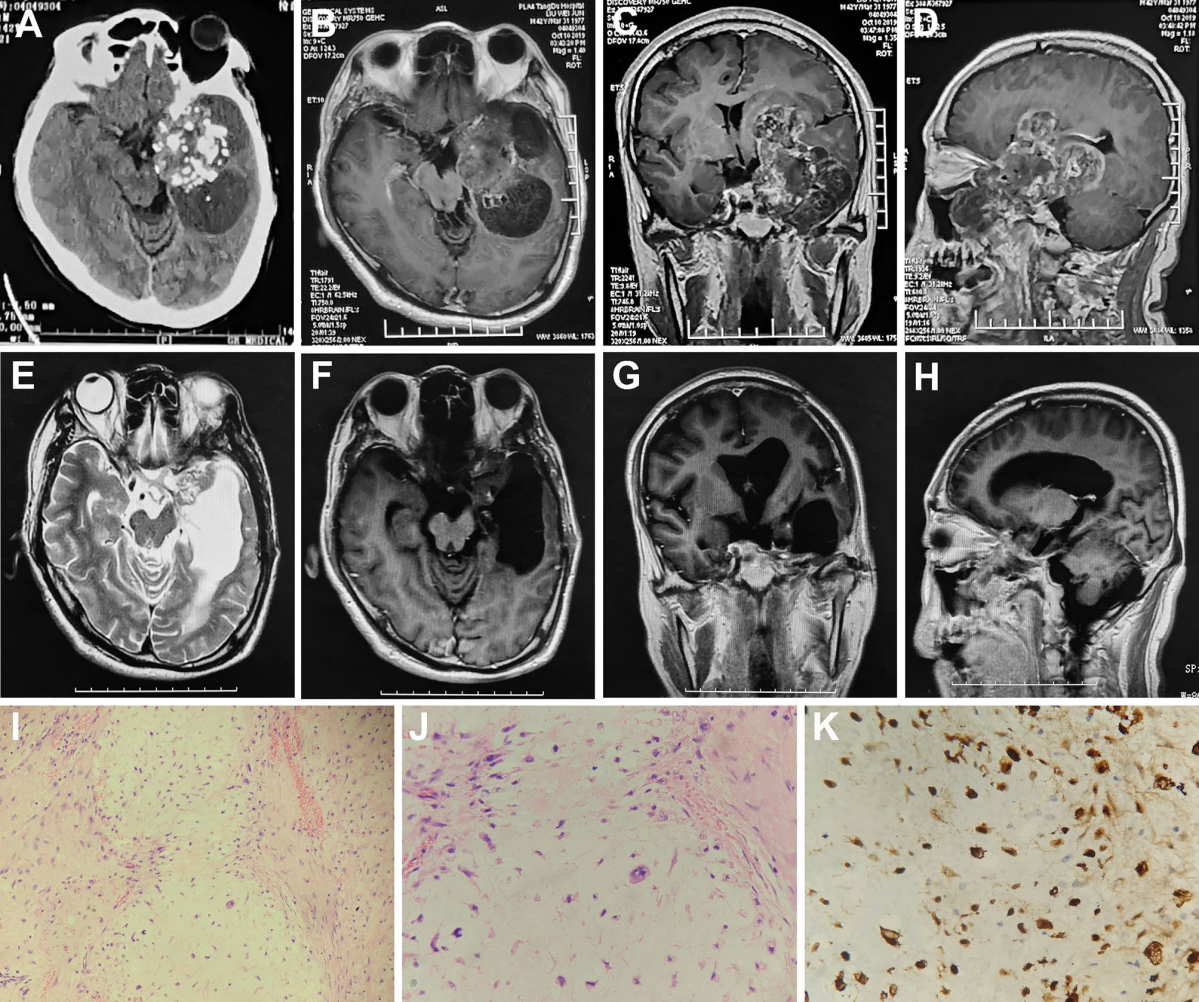
Comparison of preoperative and postoperative imaging and histopathological examination. Preoperative CT shows a patchy low-density shadow and a calcified lesion in the middle cranial fossa (**A**). Enhanced T1-weighted axial, sagittal, and coronal images show heterogeneous enhancement (**B**,**C**,**D**). Postoperative MRI T2-weighted (**E**) and enhanced T1-weighted images (**F**–**H**) demonstrate that total tumor resection was achieved. Histopathological examination at 200 × and 400 × magnification shows that tumor cells increased slightly with no clear nuclear division (**I**,**J**). Immunohistochemical staining at 400 × magnification shows S-100 positivity (+, **K**).

A 42-year-old man had blurred vision in his left eye for 4 months. The neurological examination was negative except for blepharoptosis and decreased visual acuity in the left eye. CT and MRI showed a left parasellar mass and left middle cerebral artery stenosis (Fig. 4A–D). MRI enhancement showed heterogeneous enhancement of the lesion (Fig. 4B–D). Surgery was performed through a left-expanded pterional approach. During the operation, the tumor was found to be tough, with abundant blood supply and adhesions to the anterior cerebral artery, optic nerve, and oculomotor nerve. The tumor encircled the left middle cerebral artery (MCA). The MCA was ruptured during the separation of the tumor and was microscopically sutured. Postoperative visual acuity improved, but there was no improvement in eyelid ptosis. Postoperative complications were pulmonary infection and intracranial infection. The pulmonary infection and intracranial infection were cured by physical sputum drainage, antibiotics, and lumbar cistern drainage. Postoperative MRI suggested total resection of the tumor (Fig. 4E–H). Pathological examination revealed a slight increase in tumor cells and no obvious mitoses (Fig. 4I,J). Immunohistochemistry suggested S-100 positivity (Fig. 4K). Postoperative radiotherapy was administered. There was no recurrence during 33 months of follow-up.

### Case 15

**Figure 5:**
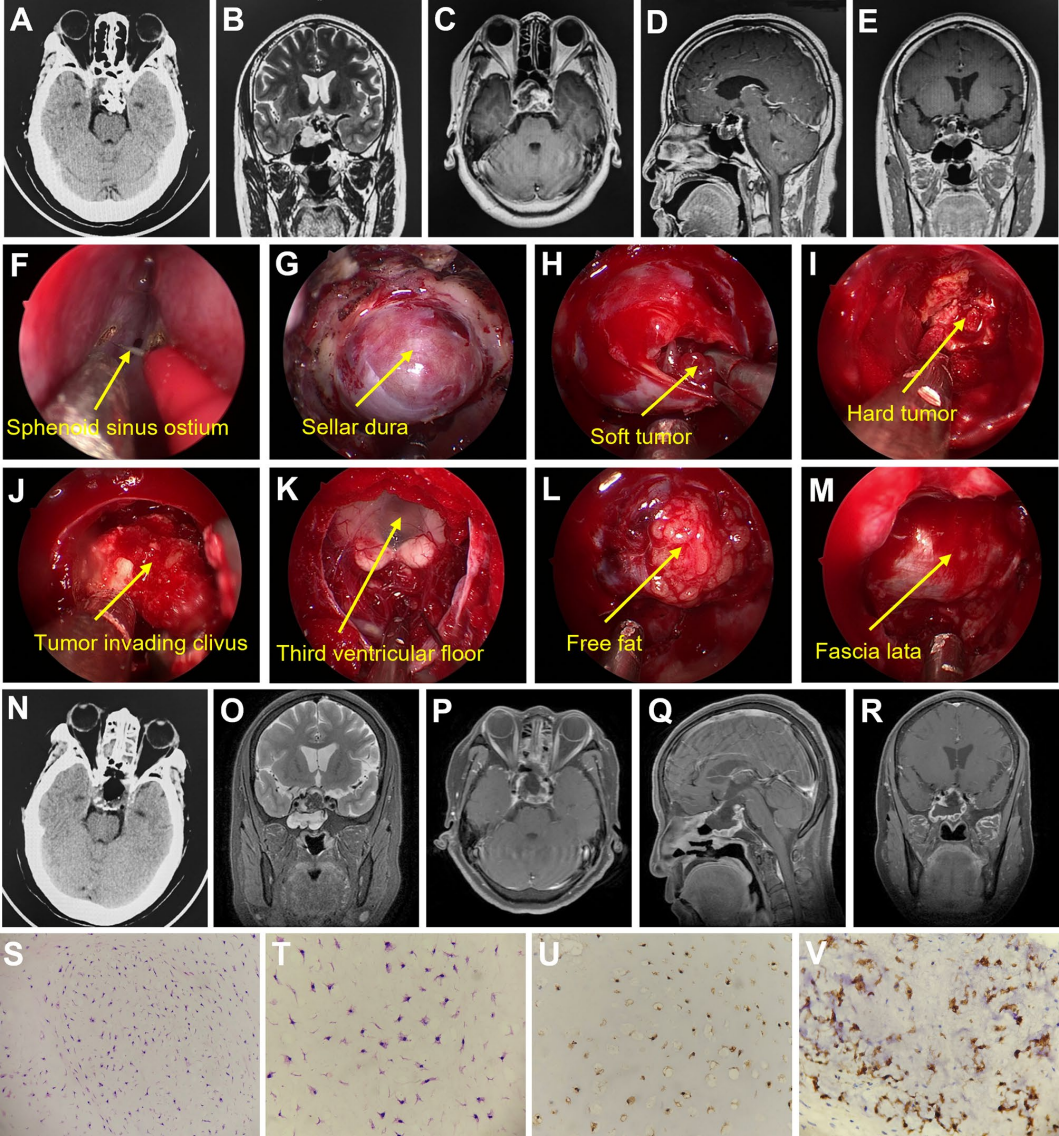
Comparison of preoperative and postoperative imaging and histopathological examination. Preoperative CT shows sellar enlargement and mixed high-low density in sellar and suprasellar regions (**A**). The lesion shows high-signal intensity in the T2-weighted image (**B**) and moderate uneven enhancement in the enhanced T1-weighted image (**C**–**E**). The sphenoid sinus ostium can be seen between the superior turbinate and the nasal septum (**F**). The dura of the sellar floor was exposed when the anterior wall of the sphenoid sinus and sellar floor bone was drilled off (**G**). Soft tumor tissue was seen after incising the dura mater (**H**). Calcified hard tumor tissue can be seen after resecting the soft tumor tissue (**I**). Removal of calcified tumor tissue located in the clival region (**J**). The mammillary bodies and the bottom of the third ventricle were observed after total resection of the tumor (**K**). The tumor cavity was filled with free fat and then covered with artificial dura matter (**L**). Finally, the operation area was covered with fascia lata (**M**). Postoperative CT and MRI demonstrated that a gross total removal was achieved (**N**–**R**). Histopathological examination at 200 × and 400 × magnification showed that tumor cells were variable in size with obvious nucleoli, and two nuclei were seen in chondrosarcoma cells (**S**,**T**). Immunohistochemical staining of SOX-9 (+, **U**) and S-100 (+, **V**), 400 × magnification.

A 56-year-old female presented with intermittent dizziness for over 10 years. She had a history of hypertension for 8 years and took oral antihypertensive drugs to control blood pressure. Physical examination showed decreased visual acuity and bilateral temporal hemianopia, and the remaining neurological findings were negative. Cranial CT and MRI showed lesions in sellar and suprasellar regions (Fig. 5A–E). MRA was used to assess the relationship between the tumor and the internal carotid artery (ICA). The preoperative diagnosis was teratoma. The tumor was resected via endoscopic endonasal transsphenoidal approach (EETA), and bilateral ICA, optic nerve, and skull base dura were exposed during surgery (Fig. 5F–K). The dura was cut open, and the mass was yellow to white with a medium blood supply. The tumor was soft in texture and eroded the slope, with ossification on the left side. After the complete resection of the tumor, the bone eroded by the tumor was removed by grinding with free fat and then covered with artificial dura matter (Fig. 5L,M). Postoperative CT and MRI showed that there was no residual tumor in the surgical area (Fig. 5N–R). Histopathological examination showed variable cell size, heteromorphic nuclei formation, and double nuclei in the cells (Fig. 5S,T). Immunohistochemistry was positive for SOX-9 and S-100 (Fig. 5U,V). The patient’s visual acuity improved significantly after the operation. There was no recurrence during the 10 months of follow-up.

## Discussion

Chondrosarcomas consists of a group of heterogeneous malignancies that most commonly occur in long bones and pelvis^8,9^. Intracranial chondrosarcoma, whose pathogenesis is associated with cartilage ossification of the skull base, is extremely rare and is most commonly found at the skull base^2^. Chondrosarcoma from extracranial sources rarely metastasizes to the brain. In our study, there were two cases with a previous history of spinal chondrosarcomas, which were considered to have metastasized to the intracranial space. Most clinical symptoms of intracranial chondrosarcoma had a slow progression, with a median duration of 6.5 months in our study. In our study, the tumors mainly invaded parasellar region, posterior fossa, middle and posterior cranial fossa, slope, and sella. The patients presented different clinical manifestations due to the compression of the cranial base nerves and blood vessels by the tumor. We found that if the lesion broke through the threshold of its own regulation, clinical symptoms of an acute attack may occur. The main clinical symptoms in our reported cases included headache (9/26), vision loss (9/26), hearing loss (4/26), blepharoptosis (3/26), abducens paralysis (2/26), facial numbness (2/26), dizziness (2/26), and diplopia (2/26).

Chondrosarcoma is invasive and destructive, and CT is often used to assess bone destruction and the relationship of the tumor to important intracranial blood vessels; MRI allows for better assessment of the relationship of the tumor to the surrounding soft tissues^10,11^. Chondrosarcoma shows a heterogeneous signal on T2-weighted images, with a “honeycomb” appearance after enhancement. However, there are no specific imaging findings in some intracranial chondrosarcomas, and they need to be differentiated from meningiomas, schwannoma, chordomas, and chondromas^7,12–14^. Based on the data from three hospitals in the past 12 years, CT showed that almost all the lesions had varying degrees of erosion and destruction, 15 cases with mixed high- and low-density shadows and 10 cases with irregular soft tissue shadows. Fifteen cases underwent CTA or MRA to determine the relationship between the tumor and peripheral blood vessels.

According to previous studies, surgery remains the preferred treatment for intracranial chondrosarcoma^15,16^. The objective of surgery is to determine the pathological nature and remove as much of the tumor as possible while preserving maximum neurological function. However, due to the complexity of the skull base anatomy, some tumors often encase or have a moderate to severe adhesion to neurovascular elements, which increases the difficulty of total tumor resection^17^. With the development of endoscopic endonasal techniques, the tumors located in the sellar or parasellar region could be resected satisfactorily. Simple endoscopic endonasal or extended endoscopic endonasal approach or endoscopic endonasal approach combined with craniotomy may help to improve the extent of tumor resection and reduce injury to surrounding neurovascular structures^18^. Because of the limited benefit, intracranial chondrosarcoma is rarely biopsied alone^19^. A summary analysis of the cases showed that GTR was performed in 13 cases, STR in 11 cases, and PR in two cases. Among the four cases that underwent an endoscopic approach, two cases underwent GTR and two cases underwent STR. Univariate Cox analysis showed that the surgical approach had no effect on OS. This was probably because the endoscopic transsphenoidal approach is a newly developed procedure and there was a small number of patients who underwent endoscopic tumor resection. However, our results showed that the extent of surgical resection was a significant variable affecting OS (HR 21.74, 95% CI 1.25–376.6, *P* = 0.03).

Since intracranial chondrosarcoma often originates from the skull base, adequate preoperative imaging can determine the relationship between the lesion and the nerves, blood vessels, and skull^15,20^. Advanced equipment, such as intraoperative electrophysiological monitoring, neuronavigation, and intraoperative ultrasound, help to improve the extent of tumor resection and minimize neurovascular injury. Additionally, considering that the tumor often erodes the bone and dura mater of the skull base, the dura mater of the skull base needs to be repaired tightly^21^. For some large tumors that invade multiple intracranial parts, one operation may not be enough to remove the tumor completely, so staged surgery or postoperative adjuvant radiotherapy is available^22,23^. In such situations, some authors advocate radical GTR, while others advocate STR combined with postoperative adjuvant radiotherapy^21,24,25^. It has been documented that the treatment of intracranial chondrosarcoma focuses on clinical symptom improvement and tumor control to maintain normal neurological function and good quality of life and minimize surgery-related complications^26^. In our study, one patient suffered intraoperative injury to the internal carotid artery, and vascular anastomosis was performed under the microscope. Due to the complexity of the skull base anatomy and the erosive characteristics of chondrosarcoma, the operation time, peritumoral tissue damage, and incidence of postoperative complications such as lung infection and nerve injury are increased^20,24,27^. In the cases presented here, postoperative complications mainly included pulmonary infection, subcutaneous hydrops, swallowing and choking, facial numbness, abducens paralysis, and intracranial infection. The cranial nerve injury was remitted to varying degrees by rehabilitation, and the remaining complications were cured by aggressive pharmacological and physical interventions.

The anatomy of the skull base is complex, and the pathology of intracranial chondrosarcoma leads to aggressive growth towards the skull base, making “complete” resection of chondrosarcoma difficult^25^. It has been suggested that even though intracranial chondrosarcoma is slow-growing and radiation-tolerant, postoperative radiotherapy can effectively control local tumor proliferation and improve survival rates^28^. Although incremental radiation doses for skull base chondrosarcoma may improve survival, they carry latent risks, particularly for the anterior visual pathway^25^. Recent studies suggest that ataxia telangiectasia and rad3 related (ATR) inhibitor and disulfiram enhance the radiosensitivity in chondrosarcoma, which may reduce radiological side effects^29,30^. The “proton” beam has also become a complementary tool after chondrosarcoma surgery^31^. At the same time, “proton” beam therapy causes less radiation damage to the surrounding brain tissue. Chemotherapy is predominantly ineffective for chondrosarcomas because of the lack of targeted therapy^32^. However, it has shown that vincristine, adriamycin and ifosfamide may be used as treatment for late stage of mesenchymal chondrosarcoma^33^. It has also been shown that brain derived neurotrophic factor (BDNF) and phosphoinositide 3-kinase (PI3K) can be used as potential targets for the treatment of chondrosarcoma^34,35^. The role of immunotherapy and antiangiogenic drugs in chondrosarcoma still needs further study. In our study, postoperative radiotherapy did not prolong OS, nor did it reduce the risk of recurrence of intracranial chondrosarcoma. This may be related to the sample size, residual tumor volume, radiation dose, tumor texture and follow-up time. It is suggested to strengthen the cooperation of multi-disciplinary team (MDT), and formulate different radiation doses according to the tumor texture and residual tumor volume.

According to the WHO classification of bone tumors (5th edition), chondrosarcoma includes four histopathological subtypes, such as classical, mesenchymal, clear cell, and dedifferentiated^36^. However, according to the 2021 CNS tumor classification, chondrosarcoma is divided into classical chondrosarcoma and mesenchymal chondrosarcoma^37^. Regardless of the anatomic location of chondrosarcoma, the classical subtype accounts for the majority of cases. Classical chondrosarcoma is classified into grades I, II, and III according to its histological morphology, lobulation, occasional calcifications, variable cell size and shape, mild heterogeneity, large and densely stained nuclei, binucleated cells, mucinous degeneration, and liquefied cartilage-like stroma, necrosis, and nuclear schizophrenia^21,28,36^. Immunohistochemical staining showed that the cytoplasm and nucleus of chondrosarcoma cells expressed S100 protein. Chordoma, chondroma, and chondromyxoid fibroma are similar to chondrosarcoma in terms of location, imaging presentation, and pathological morphology. Compared with chondrosarcoma, chondroma is more differentiated and less likely to have binucleated cells; chordoma is more likely to immunohistochemically express CK and EMA; chondromyxoid fibroma is positive for S100, and positive to desmin and CD34 to varying degrees^7,34,38^. Among our 26 cases, 24 were chondrosarcoma grade I, two were chondrosarcoma grade II, and there was no mesenchymal chondrosarcoma. Mesenchymal histology as well as tumor grade are major predictors of poor prognosis^15^. Our study is consistent with previous reports that tumor grade may be an important factor of OS (HR 131.99, 95% CI 4.05–4300.5, *P* = 0.006).

## Limitations of this study

Retrospective study bias cannot be avoided. The incidence of intracranial chondrosarcoma was low, with only 26 surgical cases in 12 years from three centers. The follow-up time was not long enough. Because of the recent development of endoscopic transsphenoidal surgery and the rarity of intracranial chondrosarcoma, only four cases were treated with endoscopic procedures in this study. Additional cases are needed to confirm the impact of endoscopic for intracranial chondrosarcoma on OS. In the next step, we plan to evaluate the impact of surgical approach and radiotherapy on OS through a systematic review combined with our cases.

## Conclusions

In this study, it was found that intracranial chondrosarcoma had a low incidence and lacked typical imaging features and was difficult to differentiate from other skull base lesions. CT and MRI could improve the diagnosis of chondrosarcoma, but the final diagnosis still needs to be confirmed by histopathology. Surgical resection was found to be the first and most important choice of treatment for intracranial chondrosarcoma. The degree of surgical excision and pathological grading were identified to affect OS.

## Data Availability

The study was retrospective. Written informed consent could not be received from all patients or guardians due to geographical and cultural differences. We obtained consent from all patients or guardians by telephone. The datasets generated during the current study are not publicly available but and are available from the corresponding author on reasonable request.
